# Morbidity Profile and Healthcare Service Utilization Pattern Among Geriatric Population in the Rural Himalayan Region of Uttarakhand, India: A Cross-Sectional Study

**DOI:** 10.7759/cureus.50000

**Published:** 2023-12-05

**Authors:** Sarika Palepu, Arkapal Bandyopadhyay, Tumul Nandan, Shradha Singh

**Affiliations:** 1 Community Medicine, All India Institute of Medical Sciences (AIIMS) Kalyani, Kalyani, IND; 2 Pharmacology, All India Institute of Medical Sciences (AIIMS) Kalyani, Kalyani, IND; 3 Community and Family Medicine, Veer Chandra Singh Garhwali Government Institute of Medical Science and Research, Srinagar, IND; 4 Dermatology, Shri Guru Ram Rai (SGRR) Institute of Medical & Health Science, Dehradun, IND

**Keywords:** healthcare seeking, adherence, chronic disease, utilization, health services, geriatric

## Abstract

Objectives: The increasing elderly population, along with their health problems, is a matter of concern, especially in the difficult terrains of the hilly Himalayan region of northern India. Hence, the present study was conducted to assess the healthcare-seeking behavior of the elderly and adherence to medication.

Methods: The present community-based cross-sectional study was conducted on 250 elderly participants by a consecutive sampling method. Data were collected during the months of July 2021 to October 2021 after obtaining institutional ethical clearance. Bivariate logistic regression was done to assess factors associated with healthcare services utilization patterns and adherence to medications in chronic diseases. Significant factors were then analyzed with a multivariate logistic regression model. Variables with p-value <0.05 on multivariate analysis were considered statistically significant.

Results: The mean age of the study participants was 67.2 (±8.09) years, and 52% were males. Chronic illness was diagnosed in 45.6% participants. Only 121 (48.4%) participants were aware of health insurance schemes among whom 95 (38%) were registered. Appropriate healthcare-seeking behavior for acute illness episodes was seen in 68.9% of participants. A government healthcare facility was the most preferred facility. Low adherence to chronic disease medication was seen in 41.2%. Participants registered under any health insurance scheme had higher adherence to medications (OR=0.36; 95% CI, 0.15-0.86; p-value=0.02).

Conclusions: The majority of the participants preferred government healthcare facilities. Registration under any health insurance scheme was found to significantly influence adherence to medications. Further qualitative studies can be of paramount importance in understanding the perspectives of the geriatric population in the study area.

## Introduction

Globally, the proportion of the population over 60 years is estimated to nearly double from 12% in 2015 to 22% in 2050. By the year 2050, the number of elderly people in the world aged 60 years and above is expected to increase to two billion from 0.9 billion in 2015. About 80% of these elderly people will be residing in low- and middle-income countries by 2050 [1].

The elderly population in India has increased from 76 million in 2001 to 100 million in 2011 forming 10% of the total population [2]. It is estimated to reach up to 150 million by the year 2020. This dependent demographic population is rising due to better control of communicable diseases, leading to better life expectancy and hence increased longevity. The population of Uttarakhand, a hilly state in north India contributed to 0.83% of the total population in India in 2011 [3]. Like other Indian states, Uttarakhand has a significant proportion of elderly people, constituting up to 7.7% of the total population in 2001 [4].

Aging leads to generalized deterioration of many organs and systems. It leads to lower effectiveness of physiological functions, accompanied by an increase in risk factors for various diseases. Hypertension and diabetes mellitus are common non-communicable diseases in the elderly and so are musculoskeletal problems such as osteoarthritis. There is also a decline in immune function causing increased susceptibility to infection and cancer increased neuronal degeneration, leading to a decline in cognitive function and dementia. Elderly patients are generally perceived to be reluctant to seek healthcare for ailments [5,6]. Hence, focus is needed as they have special needs for health and support, especially in the state of Uttarakhand, which is predominantly hilly with poor road connectivity, difficult terrain, and small scattered settlements.

Elderly people living in hilly terrain may have different perceived needs and morbidity patterns. Understanding the morbidity profile and health-seeking behavior of the elderly is essential for strengthening geriatric healthcare services delivery. There is a scarcity of information on morbidity patterns and reasons for seeking particular healthcare in hilly areas, especially in the multicultural and multiethnic localities. With this background, we planned to conduct a study to assess the morbidity pattern and analyze the healthcare-seeking behavior in the elderly population of Tehri Garhwal district.

## Materials and methods

The present community-based cross-sectional study was carried out at Madhi Chauras, Tehri Garhwal, which is the rural field practice area of the Department of Community Medicine, Veer Chandra Singh Garhwali Government Institute of Medical Sciences and Research (VCSGGIMS&R), Srinagar, India. Madhi Chauras is a part of Kirti Nagar, one of the 10 blocks of Tehri Garhwal district. This block has 154 villages comprising 10,580 houses. As per census 2011, the population of Kirti Nagar was 45,629, with 21,542 males and 24,087 females [7]. In the field practice area of community medicine, routine house-to-house visits are done by health workers. Out-patient services and laboratory investigations are also provided through the Department of Community Medicine.

Considering the prevalence (p) of appropriate healthcare-seeking behavior as 56.1% [8] and the absolute precision rate as 6.5%, the required sample size was calculated as 224. Hence, in the present study, 250 elderly individuals residing in the area, at least for the past six months and willing to participate, were approached for inclusion. Individuals who were bedridden, diagnosed with any psychiatric illness, or unable to comprehend the interview questions were excluded. The selection of villages was done based on the distance from the rural health training center (RHTC) with preferential sampling done in nearby villages. Consecutive sampling was done till the desired sample size was achieved. A list of elderly participants residing in the nearby villages was obtained from the existing family health records of the RHTC. If more than one elderly individual was present in a household, selection for inclusion in the study was done by lottery method. The study was conducted from July 2021 to October 2021.

A pretested semi-structured interview schedule was administered, and required information was obtained from the elderly individuals through house-to-house visits. The interview schedule collected information regarding sociodemographic factors, awareness about health insurance schemes, activities of daily living, self-reported acute and chronic illness profiles, healthcare-seeking behavior, satisfaction regarding recent acute morbidity treatment, and adherence to treatment in chronic diseases. Adherence to medications was assessed using an 8-point Morisky medication adherence scale (MMAS-8). Patients scoring 0 to < 6 were considered to have low adherence, 6 to <8 as medium adherence, and 8 as high adherence.

Ethical clearance was taken from the Institutional Ethics Committee, VCSGGIMS&R. Participants were interviewed after obtaining written informed consent.

The data was entered in Microsoft Excel 2013 and analyzed with the help of Stata software (version 17; StataCorp LLC, College Station, Texas). Normally, distributed data were presented as mean and standard deviation or 95% confidence interval (CI). Categorical data were presented as percentages (%). Bivariate logistic regression was done to assess factors associated with healthcare services utilization patterns and adherence to medications in chronic diseases. Variables found significant with bivariate analysis (p-value < 0.25) [9] were incorporated in a multivariate logistic regression model. Variables with p-value <0.05 on multivariate analysis were considered significant.

Operational definitions

(a). Health-seeking behavior was defined as a sequence of remedial actions taken by the person to rectify perceived ill-health [10].

(b). Appropriate or desired health-seeking behavior was defined as seeking treatment and health advice through trained doctors (both allopathic and AYUSH) from public or private health facilities.

(c). Acute illness was defined as any illness persisting for ≤ 7 days. (The acute illness profile in the present study was enquired for one month prior to data collection.)

(d). Chronic illness was defined as any illness persisting for ≥ 6 months.

## Results

The age of the participants ranged from 60 to 92 years, and the majority were males. The education status of the participants was quite varied with nearly 20% being literate up to the intermediate level. The mean years of schooling was 10 years. The majority of the participants belonged to extended family (70.4%), were staying with children and spouses (71.9%), and belonged to a lower socioeconomic status (52.4%) (Table 1).

**Table 1:** Socio-demographic characteristics of the study participants

Sl No.	Characteristic		N (%)
1	Age (in years)	Mean (±SD)	67.2 (8.09)
		Range	60-92
2	Gender	Female	120 (48)
		Male	130 (52)
3	Education status	Illiterate	37 (14.8)
		Primary school	31 (12.4)
		Middle school	43 (17.2)
		High school	37 (14.8)
		Intermediate	49 (19.6)
		Graduate	44 (17.6)
		Professional	9 (3.6)
4	Mean years of schooling (±SD)		10.0 (5.42)
5	Occupation	Currently working	7 (2.8)
		Not working	243 (97.2)
6	Monthly family income (in rupees)	Median (IQR)	10,000 (0-25,000)
		Range	0-2,00,000
7	Type of family	Nuclear	74 (29.6)
		Extended	176 (70.4)
8	Mode of stay	Staying alone	18 (7.2)
		With Spouse	37 (14.9)
		With children	15 (6.0)
		With children and spouse	179 (71.9)
9	Living arrangements	Own house	182 (72.8)
		Children’s house	66 (26.4)
		Rented accommodation	2 (0.8)
10	Socio-economic status (BG Prasad scale)	Lower	131 (52.4)
		Lower middle	17 (6.8)
		Middle	6 (2.4)
		Upper middle	92 (36.8)
		Upper	4 (1.6)

The majority of the participants were able to execute activities of daily living without any assistance. The percentage of participants who were able to go to toilet by self was 99.6 (n=249), able to eat without assistance was 98.8 (n=247), able to dress/undress properly by self was 98.8 (n=247), able to neatly dress and be well groomed was 98 (n=245), able to walk on own was 95.6 (n=239), and able to bathe on own was 98.8 (n=247).

Only 48.4% of participants were aware of any health insurance scheme, and most of them received information from family members (62.4%), followed by relatives (21%), healthcare professionals (9%), mass media (6.8%), and friends (0.8%). Registration under health insurance schemes was higher in males (41.5%) than females (34.1%). Only 88 out of 95 participants who registered for health insurance schemes could recollect the scheme name and provide appropriate documents. The majority of the participants registered for the “Ayushman Bharat” scheme (n=86), followed by the Central Government Health Scheme (n=1) and Employee State Insurance Scheme (n=1). Only 4.8% of participants have availed of any scheme in the last three months, and the most common cause was cardiac bypass surgery.

A government healthcare facility was the most preferred facility for seeking healthcare in elderly participants and their family members, followed by a private healthcare facility (Table 2).

**Table 2:** Practices regarding the utilization of a healthcare facility Percentages in parenthesis are column-wise.

Sl No.	Healthcare facility	Preference by self			Preference by family members, N (%)
		Acute illness, N (%)	Chronic illness, N (%)	Emergency, N (%)	
1	Government	129 (51.6)	206 (82.4)	209 (83.6)	208 (83.2)
2	Private	18 (7.2)	25 (10)	33 (13.2)	31 (12.4)
3	AYUSH	1 (0.4)	2 (0.8)	-	-
4	Over the counter	75 (30)	11 (4.4)	3 (1.2)	5 (2.0)
5	Self-/Home remedy	27 (10.8)	6 (2.4)	5 (2.0)	6 (2.4)

About 45 acute illnesses were reported among the study participants. A government healthcare facility was the preferred facility by 23 participants (51.1%). Appropriate healthcare-seeking behavior was seen in 68.9% of acute illness episodes. About 40 participants (89%) reported that family members accompany them to a particular healthcare facility (Table 5). Participants approaching a government health facility expressed that the main reasons for the preference were less consultation waiting time (n=11), free availability of medications (n=4), and appropriate infrastructure (n=8). Participants approaching a private health facility expressed that the main reasons for the preference were less consultation waiting time (n=6) and availability of medications within the facility (n=2). Participants taking medicines over the counter expressed that the main reasons for the preference were no consultation waiting time (n=10) and easy availability of medications (n=3).

Acute illness included headache in 12 (26.7%), fever in nine (20%), myalgia in seven (15.6%), abdominal pain in four (8.9%), and trauma/fall in two (4.4%) participants. Other causes included dental abnormalities (n=2), gastritis (n=2), urinary incontinence (n=2), visual disturbance (n=2), vomiting (n=1), cough (n=1), and breathlessness (n=1). Chronic illness was diagnosed in 114 (45.6%) participants. The mean duration of chronic illness was 6.81 (±5.3) years with a range of 0-40 years. Hypertension (n=68), diabetes (n=44), heart disease (n=9), and thyroid disorders (n=7) were the most common chronic illnesses.

Various factors affecting healthcare service utilization among study participants were analyzed with bivariate logistic regression. However, there was no significant association with any factor (Table 3).

**Table 3:** Association of healthcare service utilization with select variables among the study participants

Sl No.	Characteristic		Unadjusted OR (95% CI), P-value
1	Age (in years)		0.88 (0.77-1.03), 0.09
2	Gender	Female	1
		Male	0.52 (0.77-1.02), 0.32
3	Mean years of schooling (±SD)		0.88 (0.78-1.00), 0.046
4	Monthly family income (in rupees)		(0.99-1.00), 0.73
5	Type of family	Extended	1
		Nuclear	1.05 (0.22-4.98), 0.96
6	Socio-economic status (BG Prasad scale)	Upper middle/upper	1
		Lower/lower middle/middle	1.05 (0.25-4.33), 0.94
7	Registration under a health insurance scheme	Yes	1
		No	1.32 (0.32-5.44), 0.69

In the present study, 46 (40.3%) participants were seen to have high adherence, 21 (18.4%) were found to have medium adherence, and 47 (41.2%) were found to have low adherence (Table 4).

**Table 4:** Adherence to treatment of chronic illnesses among study participants

Sl No.	Characteristic		N (%)
1	Forget to take medicines sometimes		33 (28.9)
2	Over the past two weeks, were there any days when you did not take your medicine?		22 (19.3)
3	Ever cut back or stopped taking medication without telling your doctor, because you felt worse when you took it		34 (29.8)
4	Forget taking medicines during travel sometimes		35 (30.7)
5	Did you take your medication yesterday?		102 (89.5)
6	Feel like stopping medications when your health is under control?		39 (34.2)
7	Do you ever feel hassled to stick to the treatment schedule?		36 (31.6)
8	How often do you forget to take medications?	Never/Rarely	64 (56.1)
		Once in a while	24 (21.0)
		Sometimes	23 (20.2)
		Usually	3 (2.6)

Various factors affecting adherence were analyzed with bivariate logistic regression. On multivariate logistic regression, participants registered under any health insurance scheme were found to have significantly higher adherence to chronic disease medications (Table 5).

**Table 5:** Association of adherence with select variables among the study participants

Sl No.	Characteristic		Unadjusted OR (95% CI), P-value	Adjusted OR (95% CI), P-value
1	Age (in years)		0.97 (0.92-1.03), 0.44	-
2	Gender	Female	1	-
		Male	1.19 (0.56-2.52), 0.64	
3	Mean years of schooling (±SD)		0.97 (0.90-1.04), 0.45	-
4	Monthly family income (in rupees)		0.99 (0.99-1.00), 0.96	-
5	Type of family	Extended	1	-
		Nuclear	1.02 (0.44-2.39), 0.96	
6	Mode of stay	Staying alone	1	1
		With spouse/children	10.05 (1.17-86.53), 0.03	6.72 (0.73- 62.10), 0.09
7	Socio-economic status (BG Prasad scale)	Upper middle/upper	1	1
		Lower/lower middle/middle	0.55 (0.25-1.17), 0.12	0.54 (0.23-1.27), 0.16
8	Registration under a health insurance scheme	Yes	1	1
		No	0.37 (0.16-0.83), 0.01	0.36 (0.15-0.86), 0.02
9	Preference of health facility	Government	1	-
		Private/Informal healthcare provider/Over the counter	0.71 (0.26-1.91), 0.50	

## Discussion

This study was done to assess healthcare-seeking behavior in a rural hilly terrain, which has its own set of unique challenges, both for the healthcare providers and beneficiaries.

A characteristic finding of the present study was that most elderly participants were able to carry out activities of daily living (95.6-99.6%) without assistance. A recent longitudinal study among the elderly showed that 26.36% of older women and 20.87% of older men had low ADL scores [11]. The findings might be attributed to the exclusion of bedridden elderly in the present study.

Although 48% of the elderly participants were aware of health insurance schemes, only 38% were registered under any scheme. Registration was higher in males as compared to females. A cross-sectional study conducted in a rural area of Aligarh showed the awareness to be less in comparison to that of the present study [12]. To the best knowledge of the authors, recent data regarding the registration of elderly persons under any health insurance scheme in rural Uttarakhand are lacking, and this study finding can be of significance to the existing literature. Data on health insurance are of importance as it is a driving factor for healthcare-seeking behavior according to a study done in Maharashtra [13].

In this study, the majority of the participants preferred government health facilities to address their health needs. Few other studies also found that most elderly utilize government facilities while seeking healthcare, similar to the present study [14-16]. Another study finds that most of the participants approached unqualified persons when seeking healthcare. This may be due to the prevalent social norms and beliefs in the area of the former study [17] and/or may be due to the presence of adequate healthcare personnel at various health facilities in the present study.

In the present study, free medications at government facilities were a driving factor for the utilization of services as per the participants' responses. According to a study in rural Tamil Nadu, the high cost of care was the main reason for the non-utilization of health facilities [18]. Another study from Assam finds the financial aspect as the deciding factor for the utilization of health services [14]. Additionally, lesser consultation waiting time was another important factor for using a health facility in the present study.

The present study found headache, fever, and myalgia as the common acute conditions among the participants. Appropriate healthcare-seeking behavior was seen in 68.9% of participants for recent acute illness episodes, which is noteworthy from the perspective of a hilly terrain.

The present study found that hypertension and diabetes mellitus were the most common chronic conditions among the elderly similar to a study done in rural-urban areas of India [19]. In the present study, 40.3% of participants were seen to have high adherence, 18.4% were found to have medium adherence, and 41.2% were found to have low adherence to medications. The findings were almost similar to a study done in a tertiary care hospital wherein compliance to medications was good in 45.41%, moderate in 35.45%, and poor in 19.12% of the study participants with forgetfulness being the most common reason for poor compliance [20]. In the present study, participants with health insurance were found to be more compliant with medications.

The limitation of the present study is the possibility of recall bias as participants were retrospectively enquired about the morbidity profile and healthcare-seeking behavior. As the data were collected by a consecutive sampling method, the generalizability of the study findings is limited.

## Conclusions

In this study, the majority of the participants approached government facilities to seek healthcare. Adherence to medications for chronic diseases was low in the majority of the participants. Registration under any health insurance scheme was shown to be significantly associated with high adherence. Further qualitative research can provide greater insight into the various factors affecting healthcare-seeking behavior and medication adherence in the study area.

## References

[1] (2022). Ageing and health. https://www.who.int/news-room/fact-sheets/detail/ageing-and-health.

[2] (2022). Elderly in India - profile and programmes; 2016. https://www.mospi.gov.in/sites/default/files/publication_reports/ElderlyinIndia_2016.pdf.

[3] (2022). Uttarakhand population. https://www.census2011.co.in/census/state/uttarakhand.html.

[4] Officers of Social Statistics Division, Central Statistics Office (2011). Situation Analysis of the Elderly in India, 2011. Jun.

[5] Kirkwood TB, Ritter MA (1997). The interface between ageing and health in man. Age Ageing.

[6] Grimley Evans J (2000). Ageing and medicine. J Intern Med.

[7] (2022). Kirtinagar town population census 2011 - 2023. https://www.census2011.co.in/data/town/800298-kirtinagar-uttarakhand.html.

[8] Barua K, Borah M, Deka C, Kakati R (2017). Morbidity pattern and health-seeking behavior of elderly in urban slums: a cross-sectional study in Assam, India. J Family Med Prim Care.

[9] Bursac Z, Gauss CH, Williams DK, Hosmer DW (2008). Purposeful selection of variables in logistic regression. Source Code Biol Med.

[10] MacKian S (2003). A Review of Health Seeking Behaviour: Problems and Prospects. Manchester University of Manchester. Health Communications Systems Development Programme.

[11] Sharma P, Maurya P, Muhammad T (2021). Number of chronic conditions and associated functional limitations among older adults: cross-sectional findings from the longitudinal aging study in India. BMC Geriatr.

[12] Maroof M, Ahmad A, Khalique N, Ansari MA (2018). Health-care utilization pattern among elderly population: a cross-sectional study. Int J Med Sci Public Health.

[13] Patle RA, Khakse GM (2015). Health-seeking behaviour of elderly individuals: a community-based cross-sectional study. Natl Med J India.

[14] Hakmaosa A, Baruah KK, Baruah R, Hajong S (2015). Health seeking behaviour of elderly in rani block, kamrup (Rural) district, Assam: a community based cross sectional study. Int J Commun Med Public Health.

[15] Bartwal J, Rawat CM, Awasthi S (2016). A community based crosssectional study on self-perception of health status and health seeking behaviour among elderly population in Haldwani block, Uttarakhand. Int J Med Sci Public Health.

[16] Gupta M, Borle AL, Chhari N, Gupta S (2015). Assessment of clinico socioeconomic status and health-care support among the elderly people aged older than 60 years in urban population of Bhopal, Central India. Int J Med Sci Public Health.

[17] Qadri S, Ahluwalia SK, Ganai A, Bali S, Wani F, Bashir H (2013). An epidemiological study on quality of life among rural elderly population of Northern India. Int J Med Sci Public Heal.

[18] Ravindra Panicker P, Jerusha JD P (2019). Utilization of healthcare facilities and associated factors among rural elderly in Kanyakumari District, Tamil Nadu. Public Health Rev Int J Public Health Res.

[19] Jana A, Chattopadhyay A (2022). Prevalence and potential determinants of chronic disease among elderly in India: rural-urban perspectives. PLoS One.

[20] Shruthi R, Jyothi R, Pundarikaksha HP, Nagesh GN, Tushar TJ (2016). A study of medication compliance in geriatric patients with chronic illnesses at a tertiary care hospital. J Clin Diagn Res.

